# "Not Just a Journal Club – It’s Where the Magic Happens": Knowledge Mobilization through Co-Production for Health System Development in the Western Cape Province, South Africa

**DOI:** 10.34172/ijhpm.2020.128

**Published:** 2020-08-01

**Authors:** The Western Cape HPSR Journal Club Team

**Affiliations:** Full list of authors’ affiliations is available at the end of the article.

**Keywords:** Embedded Research, Co-Production, Knowledge Mobilisation, South Africa

## Abstract

**Background:** The field of Health Policy and Systems Research (HPSR) views researchers as active participants in processes of knowledge mobilization, learning and action. Yet few studies examine how such processes are institutionalized or consider their health system or wider impacts. This paper aims to contribute insights by presenting a South African experience: the Western Cape (WC) HPSR Journal Club (JC).

**Methods:** The paper draws on collective reflection by its authorial team, who are managerial and academic JC participants; reflective discussions with a wider range of people; and external evaluation reports. The analysis has been validated through rounds of collective engagement among authors, and through comparison with the wider sets of data, documentation and international literature. It considers impacts using a framework drawn from the co-production literature.

**Results:** Since 2012, the JC has brought together provincial and local government health system managers and academics to discuss complex systems’ and social science perspectives on health system development. The JC impacts encompass the trusting relationships (group micro-level) that have not only strengthened personal confidence and leadership skills (individual micro level), but also led to organizational impacts (meso level), such as practice and policy changes (practitioner organizations) and strengthened research and post-graduate teaching programs (academic organizations). Macro-societal impacts are, finally, judged likely to have resulted from new health system practices and policies and from academic post-graduate training activities. This set of impacts has been enabled by: the context of the JC; aspects of the JC design that underpin trusting relationships and mutual learning; the sustained participation of senior health system managers and academic managers who are able to translate new ideas into practice in their own organizational environments; and our individual and collective motivations – including the shared goal of health system development for social justice. Our challenges include risks and costs to ourselves, and the potential exclusion of challenging voices.

**Conclusion:** The principles and practice of the JC approach, rather than the JC as a model, offer ideas for others wishing to mobilize knowledge for health system development through embedded and co-production processes. It demonstrates the potential for productive human interactions to seed long-lasting systemic change.

## Background

**Key Messages**

** Implications for policy makers**

- Health policy and systems research (HPSR), rooted in complex systems perspectives, offers valuable ideas and lessons for health system development.
- Establish and sustain mechanisms of dialogue and debate with health policy and systems (HPS) researchers to generate contextually relevant and implementable ideas about health system development.
- Work with researchers to develop HPSR projects that address current health system development needs.
- Recognize that your own personal practices as well as routine processes, not just health policy documents, are mechanisms of health system development.
- Fund health system managers to participate in post-graduate health policy and system training programs, including leadership development, and so strengthen health systems.

** Implications for the public**

Researchers and policy-makers need to engage in dialogue and debate with each other, as well as other groups, to share their experiences and together generate ideas about how to strengthen health systems in ways that benefit the whole population. We report on a South African experience of bringing researchers and policy-makers together and show how simply talking to each other can generate ideas and practices that can strengthen health systems, as well as related research and university training programs. These different groups of people often work in their own worlds and do not invest energy in learning from each other. However, our experiences show that it does not take much time or effort to think together and generate new ideas that have relevance to health system strengthening. It does, however, take commitment and trust.

Health policy and systems research (HPSR) has a nearly 25-year history in low- and middle-income countries (LMICs). Its defining features are: a focus on health *systems*, rather than specific services, programs or interventions, and their complexity; concern for understanding and engaging with the political processes of policy decision-making and implementation; and a purposeful intention to support policy change.^1-3^ HPSR also acknowledges that knowledge mobilization^[1]^ is a contested and socially constructed process. Rather than being neutral arbiters of knowledge, health policy and systems (HPS) researchers are encouraged to engage in learning and action by working with health policy-makers, managers, community and civil society groups.^4,5^ The term embedded research encapsulates this idea and is promoted as an approach through which HPS researchers are *‘integrated in the ecosystem in which decision-makers operate.’*^6^

The use of research evidence, specifically, in policy-making has also become a key topic of HPSR inquiry. To date, much of the empirical literature has considered the institutional barriers to such research use in LMICs, generating ideas about how to strengthen both the processes of transferring research evidence to policy-makers and their capacities to use it.^7^ Recent studies have, however, taken a wider focus – examining, for example, mechanisms for institutionalizing knowledge use in health policy-making^8-10^ and for enabling researcher-policy-maker partnerships^11^ Knowledge mobilization for health policy-making and management is also an important, but disconnected, area of inquiry in higher income countries. In these settings there has again been growing emphasis on the types of collaborative research practices that are embraced by the term ‘embedded research.’ These include integrated knowledge translation,^12^ the co-production of knowledge in local contexts (by researchers, policy-makers, managers, practitioners, and/or service users and their families: Box 1) as well as broader collaborations and networks to share and transform knowledge.^13^ In these latter approaches, knowledge mobilization is understood as *‘the activation of available knowledge within a given context’* by those who will use it, where the boundaries between knowledge producer and user are blurred.^14^ Such collaboration is seen as particularly appropriate in addressing complex problems and systems, as combining knowledge sources (research, theory, policy and practice) is judged necessary to support system change.^15^ Recent reviews of experience from both LMIC and higher income countries settings have, however, concluded that further research is needed to understand how processes of institutionalizing knowledge use/knowledge mobilization develop over time, as well as to consider their health system and wider impacts.^7,13,16,17^

**Box 1. Key Elements of Research Co-Production^13^**

- Co-production is generally defined as ‘*a process through which inputs from individuals who are not [generally] ‘in’ the same organization are transformed into goods and services’*^18^ (cited in^13^).
- In co-production of knowledge, multiple knowledge sources are combined, usually to address a specific challenge and because they can achieve more together, than alone.
- Co-production processes ideally adhere to the following key principles: sharing of power, including all perspectives and skills, valuing the knowledge of everyone, reciprocity and building relationships.
- Outputs can be transformed by knowledge-user participation; consequently, they may better meet users’ needs and support decision-making and implementation in the local setting.

This paper seeks, therefore, to contribute to current literature by describing an experience of knowledge co-production/mobilization for health system development, from Cape Town, South Africa. An embedded HPSR hub – a collaboration between 2 universities^[2]^ – was established in 2012 to, amongst other things, draw *‘the tacit knowledge of experienced practitioners into the task of better understanding health policy and health systems, and how to strengthen policy implementation and system performance’* (CHESAI proposal 2012). To support this networking and thinking, the Western Cape (WC) HPSR Journal Club (JC) sought to be an *‘open space in which to initiate dialogue and discussion among the different HPS research groups and with practitioners about what HPSR entails and can offer’ *(CHESAI annual report 2012-2013). This paper considers the JC as a mechanism of knowledge co-production. It explores whether and how the JC has catalyzed networking and knowledge mobilization towards health system development, as well as what factors have supported and challenged it, over the period 2012-2019.

 In the next section we outline the methods used in preparing the paper. Next we describe the JC and consider its impacts, before discussing the factors enabling and challenging it and how this experience adds to the current literature. Finally, we draw out some conclusions about these experiences.

## Methods

Based on collective reflection by the authorial team, this paper presents a form of structured reflection – as also used more widely in literature on practitioner-researcher engagements.^8,19^

In May 2019 a group of 15 core JC participants, who comprise both senior public sector health managers (from the WC Government’s Department of Health and the City of Cape Town’s health department) and HPSR academics (from the Universities of Cape Town and the WC), met together over two, half days to reflect on our experience. We, first, individually and collectively interrogated our personal experiences of the JC, in part around a set of questions prompted by a recent paper on co-production.^17^ An initial summary analysis of these reflections was then prepared overnight and discussed collectively the next day, to test the initial synthesis and allow for additional insights. Finally, overall timelines of the JC and the wider activities and events into which it has fed were developed by managers and researchers separately, and then discussed collectively.

LG then led further preparatory analysis for this paper, comparing the products of the May 2019 reflections with additional material previously generated through regular JC processes of reflection with a wider range of people (see Table 1). All data were manually coded and systematically compared to allow triangulation across data sets. Whilst the May 2019 authorial judgements were largely validated through this analytic process, a few additional insights were identified (such as early concerns over the JC’s possible exclusionary nature, discussed later). A recent *‘social impact model,’* specifically developed for assessment of research co-production,^13^ was, finally, purposefully used to guide additional analysis of the JC’s impact (see below). The full draft paper was then prepared, presented and discussed with all members of the authorial team, and, finally, revised for journal submission. Although academics led the final writing process, all authors contributed actively to the surrounding conceptualization and analytic processes.

Tracking research impacts is inherently difficult, given they are diffuse and long-term.^20^ Becket et al^13^ argue that the complex nature of co-production makes the task even more difficult, and that its philosophical roots demand attention to impacts beyond traditional, tangible measures, such as peer reviewed publications. These authors suggest it is particularly important to recognize the non-linearity of co-production impacts, and how feedback loops, multiple mechanisms and interactions lead to impact over time. For example, impacts on practitioners and policy-makers as individuals occur long before papers are written and can themselves generate further impacts over time. Drawing from the realist understanding of context these authors propose a preliminary framework for mapping the different levels of impact that may result from co-production (Box 2), allowing for the partnerships and processes of co-production, as well as conceptual impacts, capacity building, and possible cultural shifts in research and practice organizations.

**Table 1:** Data Used in Preparing This Paper

**Data Set**	**Details **
May 2019 author reflections	Notes from approximately 7 hours of discussions Written notes of summary analysis of initial discussions (reviewed and revised collectively)
Reflections about the JC	Summary written notes of informal group reflections from 5 meetings: November 2013, March, October and November 2014, December 2016 (considering eg, how well JC was working, whether or not to adapt and change it, what other activities might be valuable to implement as well) (anonymized notes, available to all JC members) Notes include a report on an anonymous and short survey of JC members in 2013, about what was liked or disliked and suggestions for change
Reflections about the Think Tank meetings	Summary written notes of informal, collective reflections from 2 meetings: January and December 2017
Documentation of CHESAI	Original proposal - including plans for practitioner-researcher engagement (CHESAI proposal 2012) CHESAI annual reports 2012-2013; 2013-2014; 2014-2015 CHESAI final report 2016 (funded by the IDRC, Canada: grants no. 106788E001 and 106788E002)
CHESAI evaluation reports	Mid-term evaluation report (work undertaken November 2013-2014) (S. Soal, unpublished data, 2015) End of project evaluation report (work undertaken 2015-2016) (S. Soal, S. Spender, unpublished data, 2016) Both included specific consideration of JC, using data drawn from observations and anonymous interviews with health managers and HPS researchers

Abbreviations: IDRC, International Development Research Centre; CHESAI, Collaboration for Health Systems Analysis and Innovation; JC, Journal Club; HPS, health policy and systems.

**Box 2. The Social Impacts of Research Co-Production Work Through 4 Levels^13^**

1. Individual (micro-level) – characteristics of stakeholders, including biological and psychological aspects (ie, improved mental or physical health, improved practice and skills for practitioners).
2. Groups/networks/interpersonal relations (microlevel) – stakeholder relationships within a system (researcher/practitioner partnerships), practice changes within teams/departments.
3. Organizational or institutional (meso-level) – organizations including rules, norms (culture), capacity-building and organizational structures, funding organizations, educational institutions.
4. Societal or infrastructure (macro-level) – wider social, economic, policy and political impacts. Multiple institutions at a national scale. National public engagement, different elements of social and public value such as justice and equality.

Although this analysis was strongly shaped by the authorial team, several of those most closely involved in preparing this paper hold organizational positions in the health system and academic worlds that allow them to apply JC ideas in organizational decision-making processes and to trace those processes and their consequences. The analysis also draws on reflections from a wider range of people (see Table 1), includes comparison with wider international experience and has been developed and tested through several rounds of collective reflection and engagement. Analytic rigor was, then, derived from the iterative cycles of collective reflection, as well as data and theoretical triangulation. Finally, we include purposeful reflection on the challenges we have faced.

## Results

###  What Is the Western Cape HPSR Journal Club?

Established in September 2012, the JC is attended by provincial and local government health managers, as well as academics involved in education and research and researchers from other organizations. Meetings are held bi-monthly at a place and time agreed as convenient for most of us. From its inception, drawing from our initial discussions of HPSR as a field,^1-3^ we have had a shared interest in taking ‘*a deliberate whole systems/policy analysis/social science perspective’ *on health system development, recognizing it as a complex system (CHESAI annual report, 2012-2013). We have also purposefully used formal papers to stimulate us to share our different insights and experience on key issues, with the intention of generating shared understandings of relevance to health systems’ practice and research.

Although coordinated by a core team from the Universities of Cape Town and the Western Cape (UCT/UWC), JC paper selection (Box 3) is informed by our overall intention and by participants’ views of key issues and problems in the local health system. We have also deliberately sought to create a safe space within the JC to allow views and experiences to be shared openly (Box 3) – as the evaluator observed (S. Soal, unpublished data, 2015):

**Box 3. Journal Club Papers and Format^13^**

**Papers**

- In each meeting, 2 papers are presented and discussed in terms of their relevance to the South African and WC settings.
- Paper topics have addressed whole system change, leadership, organizational culture, accountability, learning organizations, resilience, boundary spanning, street level bureaucracy, and practice-research engagement.
- Papers include literature reviews, as well as conceptual and empirical pieces (themselves using a range of methods); and are drawn from a variety of disciplines.
- As a deliberate decision we have largely not used papers reporting South African research, in order to learn from conceptual papers and other countries’ experiences, as well as to value the knowledge of all present rather than privileging the knowledge of the participating researchers.

**Format**

- Chairs and presenters are selected to rotate formal contributions among practitioners and researchers, allowing contributions from both groups.
- Discussions are managed on a first name basis, as a signal of flattening hierarchy.
- The Chair’s role is largely to ensure all who want to contribute are able to share their thoughts and to track time.
- Sometimes the Chair also offers a wrap up of key points, but it is generally left to the individuals attending to make meaning of the discussions for their own work.

Abbreviations: WC, Western Cape; JC, Journal Club.

- *The safety of the space is not just interpersonal and professional – it also applies to the topics themselves: Space is given to explore difficult topics that are simultaneously of direct relevance to practitioners’ needs and experience, and also so new and experimental that it is hard to speak in clear messages about them*.
- *I notice that the JC is respectful, conversational and collegial. There is a tone and quality of seriousness and of listening, and transparency across the system – people are very open*.

Although the JC invitation list is now over 100 people, just over 40 people attended at least 3 meetings a year over 2012-2016 (CHESAI final report 2016). The number attending any meeting is now usually around 20-25. There has clearly been a fall-off over time in participation from researchers outside the 3 WC universities, and there is also quite variable attendance by health system middle managers. However, there has been consistent participation by the core UCT/UWC team, including younger researchers as they join, and by the senior health management team of the provincial government (WCG:H – Western Cape Government, Department of Health) as well as colleagues from the health department of the City of Cape Town municipality (CityHealth).

###  What Impacts Has the Western Cape HPSR Journal Club Had? 

**Figure 1:**
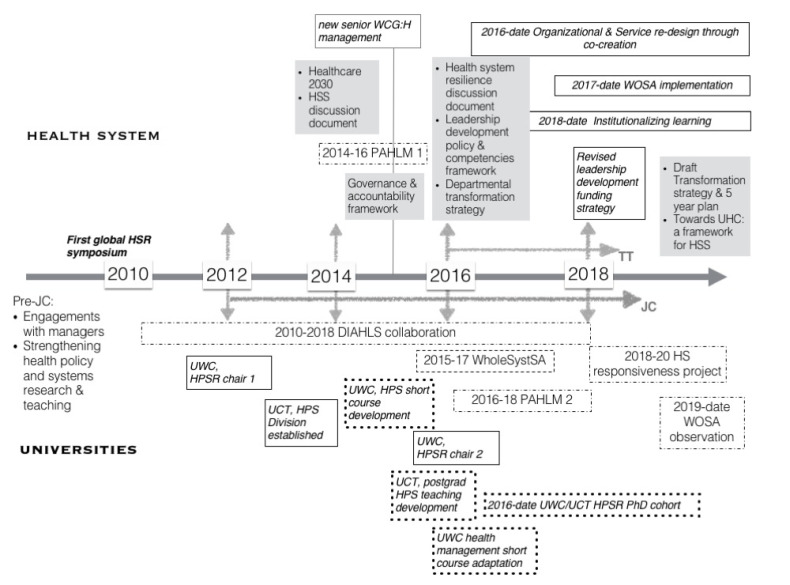
The Timeline of Key Activities Catalyzed and Influenced by the Journal Club.

Note: Blurred grey arrows = practitioner-researcher engagements (vertical arrows = Global HSR Symposia). Solid line box, italics = organizational developments. Shaded box = WCG:H policy documents. Dotted line, italics = University teaching developments. Dashed line box = collaborative HPS research and development projects (see Table 1).
 Abbreviations: WOSA, the Whole of Society Approach; JC, Journal Club; HS(S), Health Systems (Strengthening); H(P)S(R), Health (Policy and) Systems (Research); UCT, University of Cape Town; UHC, Universal Health Coverage; UWC, University of the Western Cape; WCG:H, Western Cape Government, Department of Health; PAHLM, Partnership for Health Leadership and Management.

To consider impacts we initially developed the timeline of activities that we judge have been catalyzed and influenced by the JC (Figure 1 and Table 2; see also Supplementary file 1).

**Table 2:** Collaborative Health System Development and Research Projects Linked to the WC HPSR JC

**Years**	**Project Name**	**Project Details**
2010-2019	DIALHS	An action-learning project implemented collaboratively with the WCG:H and CityHealth, to understand and support district system governance and leadership
2014-2018	PAHLM	A collaborative project to develop a leadership competency framework for public health system managers (Phase 1); extended to adapt and trial modifications to University leadership development activities, based on the competency framework (Phase 2)
2016-2019	Whole-SystSA, Whole System Change in South Africa: Understanding the experience of health system transformation in the WC province	A collaborative research project to review provincial health system development 1994-2016; also considering the lessons for monitoring future health system development
2017-date	WOSA	An approach to inter-sectoral collaboration trialed by the WCG:H in four specific areas, 2017-date; from 2019, accompanied by researcher observation
2018-date	Strengthening health system responsiveness to citizen feedback in South Africa and Kenya	A research project to consider how to strengthen system response to citizen feedback, implemented collaboratively with WCG:H

Abbreviations: WC, Western Cape; JC, Journal Club; DIALHS, District Innovation for Action Learning and Health System Development; PAHLM, Partnership for Health Leadership and Management; WOSA, the Whole of Society Approach; WCG:H, Western Cape Government, Department of Health; HPSR, Health policy and systems research.

First, as Figure 1 illustrates, 2 additional mechanisms of practitioner-researcher engagement have spun out of the JC, allowing the deepening of relationships and consolidation of ideas. We have together attended the Global Health Systems Research Symposia since 2012 – making individual presentations (2012-2018) and offering organized and satellite sessions on action-learning processes for district health system development (2012, 2016), boundary spanning and health system resilience (2016), and embedded research, collaborative governance and non-state providers (2018; see also^23^). Our collective reflections about the 2016 Symposium then led to the development of the bi-monthly ‘Think Tank’ meeting as another *‘safe, friendly, space to talk think and reflect about difficult issues outside the everyday pressures, a space whether there is mutual benefit and value-add – no deadlines, no deliverables’* (Think Tank reflection notes, December 2017). Over time, we have interrogated governance issues in this space, considered experiences of inter-sectoral collaboration in the province, and engaged with national health policy debates. Sustaining the Think Tank in 2019 proved difficult, however; perhaps because of the multiple other time demands we all face.

Second, JC relationships and ideas have, over time, fed back into a wider range of professional activities. These include a series of health system development and research projects that have been collectively developed and implemented (Table 1), a range of policy and organizational changes within the health system, educational activities and academic organizational developments (Figure 1).

We then used the social impact model (Box 2) to frame our deeper understanding of the JC’s impacts.

We judge that the group level is at the heart of the impacts achieved – where the development over time of a *‘strong, relational team’ *(May 2019 author reflections) has allowed us *‘to come together and think at the same table … grappling with theory grounded in practice’* (2013 JC member survey). Continuous engagement has allowed the deepening of relationships and emergence of shared ideas. This is a ‘*collective process of sensemaking that helps us make sense of complexity,’* as *‘the more you talk and think about ideas, the more relevance they have’* (May 2019 author reflections). In this space we have not only thought through what a systems lens is and what its implications are for health system development, but we have also worked systemically – that is, we have developed and maintained a small system founded on horizontal relationships (S. Soal, unpublished data, 2015).In both ways we have, thus, bridged the gap between academics and practitioners, research and practice, and supported wider practitioner-researcher engagement (October 2014 JC reflections; May 2019 author reflections).

The group space has also fed back into our own individual learning – with a critical and central focus on making sense in our own context of the ideas and concepts discussed. For all of us, it is a *‘complexity therapeutic circle’* where we can step outside our everyday realities to think, reflect, de-brief, re-charge and re-energize ourselves (May 2019 author reflections). The *‘discussions allow us to make sense of a complex world, providing access to conceptual lenses and models to inform practice, stimulating critical thinking’ *(Think Tank reflections, December 2017). In October 2014 within the JC, and then again in May 2019, we noted that *‘ideas and concepts have given managers the language and frames to make sense of their own experience’ *(confirmed in S. Soal, unpublished data, 2015). The discussions have also provided researchers with the language to deepen their thinking, as concepts and ideas have been tested against real world realities through engagement with managers. They have enabled entry into systems thinking concepts and experience for emerging HPS researchers, have given researchers confidence in the value of the complex/whole systems perspective and a language to connect to others (May 2019 author reflections). It also been interesting for researchers to hear about the inner workings of government and what happens at different levels of the health system – stimulating thinking about future research questions and grant applications (December 2016 reflections Think Tank). Finally, for all of us, our personal leadership practices have been influenced by the engagements – stimulating, for example, the practice of being mindful and reflective, as well as developing our skills as boundary spanners (May 2019 author reflections).

Our individual and group activities have then catalysed wider meso-level effects in our respective organizations. The personal leadership lessons derived from the JC discussions and our way of organising it, have changed our practice *‘in the spaces we hold and the way we hold them’ *(May 2019 author reflections). For example, in processes of organizational renewal and in inter-sectoral engagements to address the social determinants of health led by WCG:H (Figure 1),^21,22^ or in new CityHealth processes of engaging front-line managers and staff to develop everyday health system resilience.^23^ As noted in the October 2014 JC reflection session, *‘many of the lessons learnt from the JC are put into practice by managers … within their respective working environments’* (see also S. Soal, unpublished data, 2015).

 Our overall approach has also stimulated organizational processes that seek to institutionalize system learning. In 2017, for example, a new, regular meeting (also called a ‘Think Tank’) was established in one of the CityHealth areas as an open, managerial learning space. In 2018, the WCG:H sought to deepen system learning through quarterly ‘deep dives’ on specific issues (eg, the burden of injuries and accidents, the pressures on emergency centers) as part of routine data Monitoring and Evaluation meetings. Then in 2019 the WCG:H’s annual department-wide meeting was themed ‘connect, collaborate and learn’ for the first time, and awards were offered to recognize ‘boundary spanners’ (a term drawn from JC readings). Also, in 2019, a departmental ‘study group’ on universal health coverage was initiated to support departmental responses to current NHI policy proposals.

Finally, within the WCG:H, the ideas generated through our various engagements have fed both into wider conversations with colleagues, and into an array of provincial health system policies (Figure 1) including HealthCare 2030^24^ the current WCG:H strategic framework. The JC ideas have, thus, *‘become part of the discourse’* of the organization (May 2019 author reflections) and, through inter-linked policy documents, have informed a sustained and coherent framework that is driving and deepening organizational change over time.

The link of the JC to policy influence is clear for those involved. In interviews with department managers conducted by the external evaluator, “*When asked how JC helped members think differently, the response was ‘read Vision 2030 … it very practically brings out the interconnection between [different parts of the system]’ and it is this kind of thinking that is reinforced and supported in the JC. For one official, there is a ‘natural fit’ between JC and what the leadership of the department is trying to achieve in the way that it works, and also in its policy”* (S. Soal, unpublished data, 2015). Another example of this natural fit is the Whole-SystSA research project (Table 1), in which a joint researcher-practitioner team collaboratively reviewed WC provincial health system development 1994-2016.^25^ Not only did this project arise out of early (2013) JC discussions, but it has subsequently fed back into continuing policy development. 2019 preparatory work towards a new Departmental Transformation Strategy, then, drew 2 key lessons from it: the importance of stable, clear, distributed leadership and of instituting learning processes to support further health system development.

 In parallel, in the academic meso-level the organizational impacts of the JC have worked through 4 channels, 3 of which entail collaboration with managers:

1. The widely-known engagement with senior health system managers – including at global level through the HSR symposia – adds legitimacy to our groups and our work, with positive knock-on consequences for our organizational standing and activities. In UCT, it has helped to raise the profile of HPSR in the wider University environment, important given the newness of the field and the organizational unit leading this work. Being part of the WC HPSR network was a key element of UWC’s successful applications for 2 prestigious and nationally-funded HPSR research chair positions; and these have in turn secured further funding for research and capacity strengthening. These engagements have also given us the language and experience to talk about co-production and embedded research and its value,^26^ further legitimizing our overall research approach.
2. Post-graduate teaching programmes have been infused with HPS ideas and thinking tested through the JC (Figure 1), and also draw specifically on WC experience - both in the form of teaching cases and as our managerial colleagues teach on these programmes. The programmes are, in turn, supporting newly-appointed academic staff to develop their understanding of health systems and HPSR. Leadership development activities for public health system managers have, in particular, been informed and supported by ideas from the JC discussions and by our strengthened relationship with the WCG:H (see Box 4).
  **Box 4. Leadership Development for Public Health System Managers**
  - Based on previous research and teaching, the WCG:H initiated the collaborative PAHLM project in 2012 – through which it worked collaboratively with UCT, UWC and the University of Stellenbosch (US) to develop the 2016 Leadership Competency Framework and Leadership Development Policy (also drawing from earlier WCG:H work to support organizational culture change). In 2017-2018 the universities then self-funded a phase of work to test new approaches to leadership development based on these frameworks.
  - These activities have, in turn, influenced the universities’ teaching programs – leading, for example, to: the attachment of an additional 4-5 sessions an action learning set to the UWC short course in health management, to deepen leadership development; the use of the competency framework as a point of personal self-reflection for participants in the UCT and US PG Diplomas in Health Leadership and Management, respectively; the purposeful use of team assignments as part of the UCT student assessment approach; and the deliberate intention to, over time, recruit teams of people from the same workplace to attend the linked programs; and collective planning across the universities to ensure synergies among our programmes.
  - As our programs all actively use and engage with HPS ideas, we are supporting a growing number of managers within WCG:H (and nationally) to hold a shared, systems perspective and mindset, and to infuse this into their leadership practices.
  - Overall, then, by encouraging the participation of a critical mass of people from the same workplace and developing competence in systems thinking, our leadership development activities have the potential to make a contribution to overall system development.
  Abbreviations: PAHLM, Partnership for Health Leadership and Management; WCG:H, Western Cape Government, Department of Health; UCT, University of Cape Town; UWC, University of the Western Cape; HPS, health policy and systems.
3. The JC has directly stimulated collaborative research projects as well as Master’s and PhD dissertations (Figure 1, Table 2).
4. A range of publications involve shared authorship among those involved in the JC,^22,27^ or draw on ideas discussed in the JC.^23,26,28^

Although the macro, societal-level impacts of the JC and linked engagements are inevitably harder to discern, it can be argued that they are likely to flow from the sorts of policy and organizational changes initiated within the WCG:H and CityHealth discussed above.^16^ JC thinking is also acknowledged by managers as feeding into the wider processes of co-creating organizational and health service re-design (Figure 1), that will have long-lasting impacts on the provincial health system. Similarly, the universities’ wider teaching and leadership development activities have potential system impacts and societal level benefits (Box 4). Finally, 2018-2019 Think Tank discussions about the South African health system and NHI proposals are also feeding into our wider engagements in these debates (eg, through cross-provincial engagements with senior managers, participation in national meetings and advocacy activities).

At the same time, we have collectively contributed over time to HPSR field building – both in South Africa and globally. For those of us who are managers, global engagements offer the opportunity, beyond the JC, for ‘practice speaking ‘back’ to scholarship,’ and this was judged as: ‘*one of the critical outcomes of [our] boundary spanning approach, and one that has been especially facilitated by JC… For at least one contributor, the [2016 Global HSR Symposium] presents an opportunity to raise these questions about the relevance of academia to practice, and to speak to the necessity of up to date knowledge from the point of view of the system itself ’* (S. Soal, S. Spender, unpublished data, 2016). The academics, meanwhile, have also infused ideas generated through the JC and linked activities into our wider research and personal roles within Health System Global, the membership society for our field.

## Discussion

As the paper title notes, the experiences we report here are not just about a JC. Instead the JC is at the center of an emergent effort to bridge the practitioner-researcher divide in order to mobilize knowledge in support of health system development. Unlike the more common focus on research projects/programs in thinking about co-production^13,17^ and knowledge translation,^8^ our paper reports a systemic form of knowledge co-production^15^ occurring in and through a still-developing and small community of practicelocated within one geographical setting.^29^ It provides an example of embedded research at work, showing how co-production of ideas can inform health policy decision-making and implementation,^13,14^ as well as wider health system development,^15^ in local settings.

Ultimately, within our setting we suggest that we have had what Becket et al^13^ call paradigmatic impacts, *‘the potential to modify ways of understanding the world and shift frames of reference.’*We have seen, first, the emergence of new ideas, research approaches and relationships in this setting – such as understandings about whole system change, action-learning and the relational space we have created. These are conceptual impacts.^30^ Second, we see ‘transformative synergies’ from the knowledge we have mobilized collectively – in that it has not only generated research outputs (papers, conference presentations) and new research projects, but also led to outcomes such as health system and policy changes and capacity strengthening. The health system decision-making roles held by the practitioners amongst us have enabled instrumental impacts^30^ within the health system, the academics have fed JC ideas back into their educational activities and, together, we have fed them into new collaborative research.

**Figure 2:**
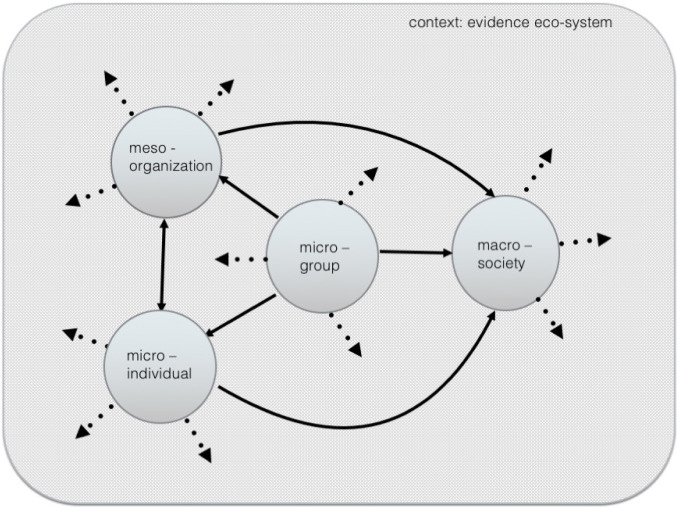
Journal Club Impacts Across Levels.
 Note: Solid lines = traceable interactions among impact levels. Dashed lines = likely interactions among impact levels. Dotted lines = untraceable spin-off impacts.

The interaction between ideas, policy and organizational changes, research developments and adapted teaching programs, is at the heart of these transformative synergies. The potential for co-production to have social impact through feedback into capacity development activities is, in particular, rarely acknowledged.^13^ *‘Ideas have legs and travel’* is the way we have explained these impacts – as well as recognizing that they have been developed and honed in a trusting, relational space: *‘it’s in the relationship between the two perspectives (manager and researcher) where the magic happens*’ (May 2019 author reflections). Our experiences (Figure 2, Supplementary file 2) demonstrate, then, how micro-level changes, at the personal and group level, can seed meso level change, including the emergence of new ideas – generating a cycle of non-linear chains of impact through which macro-level change might occur.^13^ For example: new practitioner leadership practices stimulated by JC ideas become embedded in the routines of the health system, and in turn leverage sustained system change; adapted leadership development programs draw on new knowledge honed in the JC and then seed the spread of micro-level changes in their participants, with wider system effects (Box 4); and the practice of co-production embodied in the JC introduces emerging researchers to systemic understandings and relevant research methods, which feed back into their research and training activities, spreading the learning further.

The factors that have supported the JC engagement and impacts include the context in which we are located - characterized by prior relationships^[3]^ based, as is common in communities of practice,^29^ on mutual respect, and an evidence ecosystem open to learning.^25,31^ Our individual and collective motivations for engaging in the JC are also important, as in any practice community.^29^ Through the JC, the academics among us set out to deepen our learning about health systems through engagement with practitioner colleagues. We recognized the limits of our formalized, research knowledge in understanding the everyday realities of complex health systems and how to strengthen them, and sought to cross the bridge between research and practice (S. Soal, S. Spender, unpublished data, 2016). In parallel, valuing the academics’ HPSR perspectives, those of us who are health system managers set out to engage intellectually to deepen our practice for health system development. Collegial engagement around ideas was then the defining characteristic of the experience.^21^ Recognizing that *‘to change the world, we must first change ourselves’* (May 2019 author reflections) we all value the space to reflect together and the learning about system complexity we have gained from the JC. We see it as contributing to our personal leadership and practice/research journeys and as supporting our shared political project, to strengthen the health system towards health equity and social justice. Shared purposes^13^ are critical for co-production, and political rationales may be most important.^17^

Specific aspects of the JC’s design have also enabled our engagements and impacts (Box 5). Some design features reflect wider recommendations for traditional academic JCs.^32,33^ However, our key focus has been on considering the relevance of the papers to health system practice rather than scientific skill development. More importantly, these features reflect core principles acknowledged to support co-production – such as equality among participants, power sharing, valuing all forms of knowledge and relationship building.^13,17^ “*Practitioner contributors refer to the freedom and absence of ‘fear’ in JC, to its ‘lack of formality and hierarchy,’ to the fact that you can ‘focus on ideas’ and ‘discuss these openly” *(S. Soal, unpublished data, 2015).The way we have managed our collective spaces has, then, enabled our collective thinking, sustained our relationships and supported wider impacts. As the evaluator commented, *“the networked character’* of our engagement *‘creates an approach to HPSR promotion that is not located in any single organization, or institution, yet is organizational in that it galvanizes the energies and focus of people into common intellectual and practical pursuits” *(S. Soal, unpublished data, 2015).In essence, then, as would be expected of a community of practice,^29^ the JC has created a *‘social context’* in which *‘knowledge, practice and policy can be interrogated, modified and learned’*^13^ and transformed into shared ideas, knowledge and wider action. It represents a systems’ model of knowledge to action,^15^ and illustrates how cultural-cognitive factors can support the institutionalization of knowledge use.^16^

**Box 5. Features of Journal Club Design and Approach That Support Impacts**

- The mutually agreed ‘systems’ focus.
- Papers seen as an accepted, credible and neutral catalyst for reflection and conversation, that act as a focusing point outside researcher/manager worlds.
- The deliberate and careful selection of papers that challenge our world views and stimulate our thinking, speak to current health system realities and generate ideas that can be picked up in our personal leadership practice.
- Focusing on the real-world relevance of the ideas presented in the papers, drawing on tacit and formal knowledge, rather than on scientific critique based primarily on so-called ‘expert’ knowledge.
- The creation of a non-threatening and (largely) non-hierarchical, safe space through specific practices that nurture trusting relationships, such as sharing roles, respectful engagement and ensuring ‘*you don’t leave with a to do list’* that only adds to your usual workload (May 2019 author reflections).

The long-term and sustained engagement of a central core of managers and academics has, crucially, allowed us to maintain our purpose and focus, even as a wider array of people have shifted in and out of the JC. It has supported the maturation of ideas through cycles of engagement that have spun off into wider conversations and activities. The involvement of senior health system managers is unusual in wider co-production experience^13,34^ and has clearly been critical in feeding JC ideas into health system policy and practice. In addition, by supporting the JC and linking JC ideas to wider educational and research activities, the academics have demonstrated the broad range of researcher entrepreneurship that facilitates policy impact.^19^

 However, sustaining our activities is challenging and we recognize risks in the way we have engaged. Whilst we feel that our foundation of relationships makes it possible to ‘talk truth to power’ within the collective, we recognize that concern for the relationships may silence contestation around ideas (May 2019 author reflections). The more powerful voices among us may also crowd out others, despite our intentions (JC notes November 2013), and we risk being exclusive in participation (JC notes November 2014; S. Soal, S. Spender unpublished data, 2016) and through our shared language (May 2019 author reflections). By holding the space specifically for those who want to develop and deepen a systems perspective, we are perhaps missing opportunities to infuse ideas into more traditional health science networks and thinking. Finally, despite our intentions, we have not been able to engage a wider range of system actors - such as middle managers and front-line health staff and civil society organizations (JC notes November 2013; November 2014; December 2016).

The risks of co-production outlined by Oliver et al,^17^ therefore, have resonance for us. Amongst others, it requires particular inter-personal skills and is seen as *“time-consuming, ethically complex, emotionally-demanding, inherently unstable, vulnerable to external shocks, subject to competing demands and expectations”*^35^ (cited in^17^). For academics, it can be seen as threatening our neutrality and independence and, despite our own organizations’ concerns for socially responsive activities, is not always well recognized in academic career development. For health system managers, it can be seen as a luxury activity in the face of the immediate need to manage the huge pressures facing the health system.

 Finally, we recognize that in some ways we have been more successful in working with the global HPSR community than the South African health systems and health research community. In 2019 we sought, then, to engage in the wider South African NHI debates by applying systemic perspectives and planned to strengthen such engagement in the future.

## Conclusion

 The JC does not have a lifetime-guarantee and will only continue for as long as members see value in it as part of a shared approach to systemic learning.

We also do not suggest that this approach to knowledge co-production can simply be replicated: we are a particular group of people, engaging in a particular space at a particular time. Nonetheless, we suggest that the principles and practices of our approach have wider relevance – and hope they will live on through our personal and wider organizational activities. Our experiences point to the potential for knowledge to have impact through a systemic practice of engagement informed by an understanding of organizational complexity. It is, in essence, a systemic model of knowledge to action^15^ that shows *‘the potential for the research process and productive human interactions to affect much deeper and more enduring change.’*^13^ Such an approach to knowledge mobilization requires a critical mass of interested participants willing to take the risk of crossing over established boundaries and enabling feedback loops, in pursuit, in our case, of the shared goal of strengthening health systems for social justice.

 Ultimately, our experiences suggest that:

- HPSR ideas and collective sense-making processes can support health system development;
- Relational and trustworthy spaces of engagement between managers and researchers who have shared goals can be effective places of knowledge mobilization to support action;
- Long-term and consistent engagement among stable groups of managers and researchers enables knowledge mobilization and impact;
- The mandated policy and organizational leadership roles of managers provide mechanisms for meso- and macro-level research impacts;
- The mandated teaching and capacity development roles of universities, together with their research role, enable the spread of ideas through people and organizations into society.

## Acknowledgements

 We thank all colleagues who attend the Western Cape HPSR Journal Club for their continuing engagement.

 The Western Cape HPSR Journal Club team that developed this paper consists of the following members, displayed with their affiliations:

 At Health Policy and Systems Division, School of Public Health and Family Medicine, University of Cape Town, Cape Town, South Africa: Leanne Brady, Lucy Gilson, Jill Olivier, Marsha Orgill, Maylene Shung-King, Eleanor Whyle.

 At Department of Health, Emergency Medical Services, Western Government, Cape Town, South Africa: Leanne Brady.

 At Department of Health, Western Cape Government, Cape Town, South Africa: Keith Cloete, Beth Engelbrecht, Krish Vallabhjee.

 At Department of Health, City of Cape Town Municipal Authority, Cape Town, South Africa: Soraya Ellokor.

 At School of Public Health, University of the Western Cape, Cape Town, South Africa: Asha George, Uta Lehmann, Ida Okeyo, Nikki Schaay, Helen Schneider.

 At Department of Global Health and Development, London School of Hygiene and Tropical Medicine, London, UK: Lucy Gilson.

## Ethical issues

As noted in the methods section we did not seek ethics clearance for the processes of collective reflection on our *own* experience that generated data used in this paper. However, ethics clearance was granted for the CHESAI evaluation on which we also draw by the University of the Western Cape, South Africa (registration number HS/16/6/40).

## Competing interests

 LG reports that the authors of the paper are all participants in the activity discussed.

## Authors’ contributions

 All authors contributed to this paper by generating, sharing and collectively discussing their own reflections and insights, and by reviewing the draft and final versions of the paper. LG coordinated the writing process, including conducting additional analyses reviewed by the full authorship team.

## Funding

 The preparation of this paper received no funding. The Journal Club was originally developed as part of the Collaboration for Health Systems Analysis and Innovation, funded by the International Research and Development Centre, Canada (grants 106788E001 and 106788E002).

## Endnotes

 [1] Multiple terms are used in the literature on knowledge transfer, translation, exchange and mobilization; including, in LMICs, the funder-inspired terms of GRIPP (getting research into policy and practice) and research uptake. In many instances this literature focuses specifically on knowledge as research evidence, and adopts a linear, or one-way, model of understanding the process of transferring this knowledge into policy and practice. In this paper we use the term knowledge mobilization to recognize the multiple, dynamic pathways through which the combination of multiple knowledge sources (including concepts and ideas, practice or tacit knowledge, rather than only research evidence) bring about change in policy and practice. We understand knowledge mobilization in this sense, to be central to understandings of embedded research and knowledge co-production process. [2] Initiated in 2012, the Collaboration for Health Systems Analysis and Innovation (CHESAI) sought to develop an HPSR hub in Cape Town, rooted in concern for health system equity and in social science perspectives on health policy and health systems. Led by the HPSR groups within the UCT and the UWC, CHESAI built on their existing HPS teaching and research activities and wider engagement with managerial and practitioner colleagues provincially and nationally. [3] Trusting relationships had been established through activities such as: the UWC Winter School, a continuous professional development programme aimed at South African public health managers, which has run annually for over 25 years; UCT’s Oliver Tambo Fellowship programme, which has provided health management training to public sector managers since 1996; and the District Innovation and Action Learning for Health System development collaboration, 2010-2019.

## Supplementary files


Supplementary file 1. The Timeline of the WC HPSR Journal Club and Linked Activities.

Supplementary file 2. The Social Impact of the WC HPSR Journal Club (Using the Framework of^13^).
