# Supplementary material for: "Not Just a Journal Club – It’s Where the Magic Happens": Knowledge Mobilization through Co-Production for Health System Development in the Western Cape Province, South Africa
Source: Int J Health Policy Manag. 2020 Aug 1;11(3):323–33. doi: 10.34172/ijhpm.2020.128 (PMC9278475; doi:10.34172/ijhpm.2020.128)
Supplement: Supplementary file 1 — The Timeline of the WC HPSR Journal Club and Linked Activities. [file ijhpm-11-323-s001.pdf]

Supplementary file 1. The timeline of the Western Cape HPSR Journal Club and linked activities

		**Activities beyond Journal Club**	
	**Journal Club (and Think Tank)**	**Academics**	**Managers**
**PRE-CHESAI**<br><br>2008-2011		*Strengthening of HPS and leadership post-graduate training activities at HEIs*	
		Managerial involvement in UCT post-graduate teaching (2009-2013)<br><br>***WCG:H* commissions researchers to conduct evaluation work**<br><br>***Collaborative* development and initiation of new action-learning research project with WCG:H and CityHealth (the DIAHLS project, 2010-2018)**	
		First HSR Symposium, Montreux (2010)	
**CHESAI start**<br><br>2012	Journal Club initiated:<br>With discussion about what HPSR is and the case study method of HPSR	***UWC, awarded SARChI HPSR Chair 1***	
		Collaborative: Second HSR Symposium, Beijing	
2013	Bi-monthly Journal Club, with themes of: systems change;, maternal health; leadership; organizational culture; & practice-research engagement.<br><br>*End of year reflection:* decision to continue with focus on issues of relevance to new provincial health strategy		
2014	Bi-monthly Journal Club, with themes of: WCG:H HealthCare 2030 vision; policy implementation; quality of care; community accountability<br><br>*March open discussion around HC2030*, research needs & priorities<br><br>*October open discussion*, reflection on HSR symposium	***WCG:H initiated:* collaborative leadership & management development activities with HEIs (the PAHLM project), phase 1 competencies (2014-2015)**<br><br>*Collaborative*: Third HSR Symposium, Cape Town	

		Activities beyond Journal Club	
	**Journal Club (and Think Tank)**	**Academics**	**Managers**
		UCT, HPS Division established	WCG:H POLICY DOCUMENTS:<br>• Healthcare 2030: the Road to Wellness (strategic framework)<br>• Conceptualizing the Western Cape health system: implications for system design, planning & strengthening (HSS discussion document)
2015	<u>Bi-monthly Journal Club</u> with themes of: community accountability, programs & systems, evidence to practice, non-state providers, systems transformation	*Additional HPS short courses offered collaboratively and annually by UCT/UWC within UWC Winter School continuing professional development program (ongoing)*	WCG:H POLICY DOCUMENTS:<br>• Governance & Accountability framework<br><br>***WCG:H: new senior leadership team appointed***
		Collaborative: **Whole-SystSA project initiated with WCG:H (2015-2017)**	
2016	<u>Bi-monthly Journal Club</u> with themes of: resilience, time &history in HPS research & practice, mental health, plural health, learning organizations<br><br>*December reflection discussion*	*UCT, Refreshed leadership development post-graduate training program (drawing on PAHLM project thinking)*<br><br>*UCT, additional MPH HPS course introduced*<br><br>*UWC/UCT, initiation of HPSR PhD Cohort*<br><br>***UWC, awarded SARChI HPSR Chair 2***	WCG:H POLICY DOCUMENTS:<br>• HS Resilience framework<br>• Departmental Leadership Development Strategy<br>• Leadership Competency Framework<br><br>***WCG:H organizational development & service re-design co-creation processes (2016-date)***
**CHESAI ends**		*Refreshed managerial involvement in UCT post-graduate teaching, 2016-date*<br><br>*Collaborative:* Fourth HSR Symposium, Vancouver	

<table>
<tr><th></th><th></th><th colspan="2">Activities beyond Journal Club</th></tr>
<tr><th></th><th>Journal Club (and Think Tank)</th><th>Academics</th><th>Managers</th></tr>
<tr><td rowspan="2">2017</td><td><u>Bi-monthly Journal Club</u> with themes of: SLBs & accountability/boundary spanners, HS responsiveness, system evaluation & learning organizations, public value, intersectoral action for health

<u>Bi-monthly Governance Think Tank</u>: planning, governance perspectives, mapping district interfaces, hospital level, support service interface, reflection (Jan planning; Dec reflection)</td><td>UWC, Winter school management course adapted to include action learning set (drawing on PAHLM project thinking)</td><td>WCG:H POLICY DOCUMENTS:
• Transformation Strategy

WCG:H WOSA implemented (2017-date)

CityHealth Area South implements Think Tanks, as managerial learning space</td></tr>
<tr><td></td><td colspan="2">HEIs initiated: collaborative leadership & management development activities (the PAHLM project), phase 2 pilot implementation with WCG:H
(2017-2018)

Collaborative: health system resilience research with CityHealth (2017-2018)</td></tr>
<tr><td rowspan="2">2018</td><td rowspan="2"><u>Bi-monthly Journal Club</u> with themes of: intersectoral collaboration, social determinants of health, research-practice engagement, UHC reform (Thai, Brazil, S Korea), HS contracting/purchasing

<u>Bi-monthly Governance Think Tank</u>: WOSA, governance, South African HS ‘crisis’, NHI</td><td colspan="2">WCG:H initiated: revised funding principles for leadership and management development activities

HEIs initiated: Research project on health system responsiveness (2018-2020)

Fifth HSR Symposium, Liverpool</td></tr>
<tr><td></td><td>WCG:H Purposeful engagement about health system development with other provincial depts of health

WCG:H new forms of learning - monitoring and evaluation Deep dives</td></tr>
<tr><td>2019</td><td><u>Bi-monthly Journal Club with</u> themes of: learning systems, community responsiveness, boundary spanners, purchasing</td><td>Purposeful, wider engagement about NHI and health system development through social media and other fora</td><td>WCG:H POLICY DOCUMENTS
• Draft Transformation Strategy and five year plan</td></tr>
</table>

		**Activities beyond Journal Club**	
	**Journal Club (and Think Tank)**	**Academics**	**Managers**
	Bi-monthly Governance Think Tank: reflection on the Journal Club, for this piece, May 2019		• Towards UHC: A framework for action, Health System Strengthening (approved)<br><br>***WCG:H Provincial indaba emphasizes learning; establish UHC study group***<br><br>WCG:H Purposeful engagement about health system development with other provincial depts of health
		WCG:H initiated: **UWC engagement in reflection about WOSA**	

Abbreviations:

CHESAI, Collaboration for Health System Innovation and Analysis
CityHealth, health department of the City of Cape Town
DIALHS, District Innovation for Action Learning and Health System development (research project)
NHI, National Health Insurance
PAHLM, Partnership for Health Leadership and Management
UCT, University of Cape Town
UWC, University of the Western Cape
WCG:H, Western Cape Government, Department of Health
Whole-SystSA, Whole System Change in South Africa: Understanding the experience of health system transformation in the Western Cape Province
WOSA, Whole of Society Approach to inter-sectoral collaboration
HEIs, higher education institutes
HS, health system
NHI, national health insurance
UHC, universal health coverage

Key to text in 'Activities beyond Journal Club':

- Plain = wider engagements
- Italics = post-graduate teaching activities
- Bold = collaborative research and development projects
- Bold & italics = organizational changes
- Capitals = WCG:H policy documents
