# Supplementary material for: "Not Just a Journal Club – It’s Where the Magic Happens": Knowledge Mobilization through Co-Production for Health System Development in the Western Cape Province, South Africa
Source: Int J Health Policy Manag. 2020 Aug 1;11(3):323–33. doi: 10.34172/ijhpm.2020.128 (PMC9278475; doi:10.34172/ijhpm.2020.128)
Supplement: Supplementary file 2 — The Social Impact of the WC HPSR Journal Club (Using the Framework of 13 ). [file ijhpm-11-323-s002.pdf]

Supplementary file 2. The social impact of the Western Cape HPSR Journal Club (using the framework of [13])

<table>
<tr><th></th><th>Academics</th><th>Managers</th></tr>
<tr><td>Individual level (micro)

Characteristics of stakeholders including biological and psychological aspects</td><td>• Enabled entry into systems thinking ideas and concepts
• Fed into own continued, wider ‘thinking work’ and research ideas (eg, marginalised services, ways of learning)
• Grounded our thinking in real world & everyday health system realities, allowed theoretical ideas to be tested real world complexity and challenges - demonstrating the substantive relevance of research topics
• Generated greater understanding of the complex, real world challenges associated with system development eg, community participation, and system priorities and realities</td><td>• Informed our own ideas – given us language/frames to make sense of our experiences
• Stimulated our consciousness, stimulated us to be mindful
• Personal leadership development</td></tr>
<tr><td>Group (micro)

stakeholder relationships within a system</td><td colspan="2">• We have developed a strong relational team, by consolidating and strengthening trusting relationships, where tacit and formal knowledge is valued
• We have generated shared understandings and collective sense-making around the systems lens – around what thinking and working systemically entails and how it adds value to health system development
• We have validated the system and complexity lenses and perspectives as having practical relevance (set against other dominant research discourses eg, implementation science)
• We have consolidated and accumulated understanding and sense over time</td></tr>
<tr><td>Organizational (meso)

organizations including rules, norms (culture), capacity-building and organizational structures</td><td>• Raised profile of HPSR groups in own organizations - supported eg, UWC HPSR chairs, development of UCT HPS division
• Legitimated co-production research approach
• University teaching programmes infused with systems thinking, support wider leadership development
• Fed into wider research activities
• Fed into papers
• Supported own presentations/roles in HSR symposia
• Contributed to our ‘social responsiveness’ activities, as researchers in HEIs</td><td>• In WCG:H/CityHealth applied ideas in our practice, in our engagement with others inside and outside our organization, so have had impact on the management and leadership of people and services
• In WCG:H, ideas have become part of the discourse of the organization (eg, resilience, systems thinking, boundary spanning); have influenced senior leadership thinking about organizational development towards social justice/health as a right (eg, the Whole of Society Approach - WOSA); have influenced organizational practices (eg, recognising boundary spanners, working towards a learning organization)
• In WCG:H/CityHealth: connected to experienced researchers and their</td></tr>
</table>

	**Academics**	**Managers**
		‘resources’, including international experience through HSR symposia
	• Academics engaged practitioners in teaching<br>• Practitioners engaged academics in practice spaces<br>• Collaborative research projects/engagement of mutual benefit developed and implemented (eg, the Whole-Syst SA project; the PAHLM; the CityHealth resilience work; the Responsiveness project; engagement with WOSA, and Emergency Medical Services)	
Societal (macro)<br><br>*Wider social, economic, policy and political impacts. Multiple institutions at a national scale. National public engagement, different elements of social and public value*	• Fed into wider national & international research<br>• Fed into our teaching activities, which have national and international participants<br>• Fed into national HPSR field-building activities eg, through Public Health Association of South Africa<br>• Fed into global HPSR field building eg, through roles in Health Systems Global and HSR Symposia	• Fed into wider conversations with other provincial heads of department/public health leaders across the country, about the value of ‘systems thinking’/ of a systems perspective for their work eg, through practical efforts to engage collaboratively across provinces in response to the current NHI debates<br>• Fed into engagement in HSR Symposia
	• Our collective thinking has included how to engage more widely within South African health policy and system debates and advocacy	
Sources: CHESAI annual reports 2012-13, 2013-14; notes of JC reflections (November 2013; March 2014; October 2014); May 2019 author reflections		
